# O-GlcNAcylation in diabetic cardiac remodeling and a proposed endothelial-macrophage-fibroblast network

**DOI:** 10.3389/fimmu.2026.1976141

**Published:** 2026-09-22

**Authors:** Jin Xu, Yan Li, Yan Zhang, Juan Feng, Yijun Wei, Fei Wang

**Affiliations:** 1 Department of Endocrinology, The 940th Hospital of Joint Logistic Support Force of Chinese People’s Liberation Army, Lanzhou, Gansu, China; 2 Department of Cardiovascular Medicine, The 940th Hospital of Joint Logistic Support Force of Chinese People’s Liberation Army, Lanzhou, Gansu, China; 3 Lintao County People’s Hospital, Dingxi, Gansu, China

**Keywords:** cardiac fibrosis, diabetic cardiac remodeling, endothelial metabolic memory, hexosamine biosynthetic pathway, immunometabolism, macrophage immunometabolism, microvascular dysfunction, O-GlcNAcylation

## Abstract

Diabetic cardiac remodeling involves microvascular dysfunction, inflammatory myeloid responses and fibroblast activation. O-linked beta-N-acetylglucosamine (O-GlcNAc) modification connects nutrient metabolism to protein function within each compartment, but its role in coordinating communication between them remains a hypothesis. This Mini Review evaluates that hypothesis against the available evidence. Diabetic models support causal effects of endothelial O-GlcNAc perturbation on coronary dysfunction, and an arterial endothelial CaMKIIalpha–small-extracellular-vesicle circuit provides evidence for persistent communication with cardiomyocytes after glucose normalization. Macrophage substrate mechanisms and fibroblast contractile responses are drawn partly from other organs or non-diabetic disease. Their relevance is therefore assessed separately from experimental design and human association. We compare O-GlcNAc with parallel AGE–RAGE, oxidative-stress and nutrient-sensing mechanisms, identify limitations of antibody-based and site-level measurements, and address sex as a biological variable. The synthesis supports O-GlcNAc as a candidate intracellular mediator of persistent multicellular remodeling, rather than an established intercellular coordinating signal. A consolidated experimental strategy tests whether endothelial O-GlcNAc is necessary for myeloid recruitment and subsequent fibrosis under matched glycemic conditions, and whether defined endothelial cargo rescues those effects. Cell-, substrate- and time-resolved interventions will be needed to separate pathogenic persistence from adaptive O-GlcNAc responses.

## 1 Introduction

Diabetes increases the risk of heart failure even when obstructive coronary disease or hypertension does not fully explain cardiac dysfunction. The diabetic myocardium is therefore often described through metabolic inflexibility, oxidative stress, calcium mishandling and fibrosis. O-GlcNAcylation has entered this narrative as a nutrient-sensitive post-translational modification that is chronically elevated in experimental diabetes and human diabetic myocardium (1). Yet a cardiomyocyte-centered account misses an immunological problem: microvascular endothelial cells, recruited monocytes, tissue macrophages and fibroblasts exchange signals before fibrosis becomes an irreversible organ-level phenotype. Recent immunology-focused syntheses of diabetic cardiomyopathy likewise identify endothelial and myeloid activation as central determinants of remodeling (2).

O-GlcNAcylation denotes the reversible addition of O-GlcNAc to serine or threonine residues on nuclear, cytosolic and mitochondrial proteins. O-GlcNAc transferase (OGT) adds the sugar and O-GlcNAcase (OGA) removes it. We use O-GlcNAc modification for this chemical event, O-GlcNAc abundance for a measured signal, and O-GlcNAc signaling only for a supported downstream functional consequence. Because two enzymes regulate thousands of substrates, total abundance is a poor surrogate for the action of one cell or protein (3). Acute elevation can support stress adaptation, whereas chronic or mistimed modification can sustain pathogenic transcription, metabolism or proteostasis (4). Circadian phase, nutrient state, enzyme localization and competing phosphorylation further shape these effects (5).

We evaluate a proposed endothelial–macrophage–fibroblast network, with cardiomyocyte stress and matrix remodeling as associated outcomes (**Figure 1**). Gray dashed arrows and question marks identify intercellular links for which a causal role of O-GlcNAc remains unproven. Its contribution is an evidence map that distinguishes cell-autonomous O-GlcNAc effects, demonstrated intercellular communication and missing causal connections. O-GlcNAc is primarily an intracellular modification; ligands, vesicle cargo and altered endothelial adhesion would transmit its consequences between cells. Current studies do not establish that endothelial O-GlcNAc initiates macrophage-dependent fibrosis in a single diabetic cardiac model, or that it coordinates the network more specifically than other diabetic mediators. We therefore treat this integrating role as a falsifiable hypothesis and assess experimental design separately from disease and organ relevance (**Table 1**).

**Figure 1:**
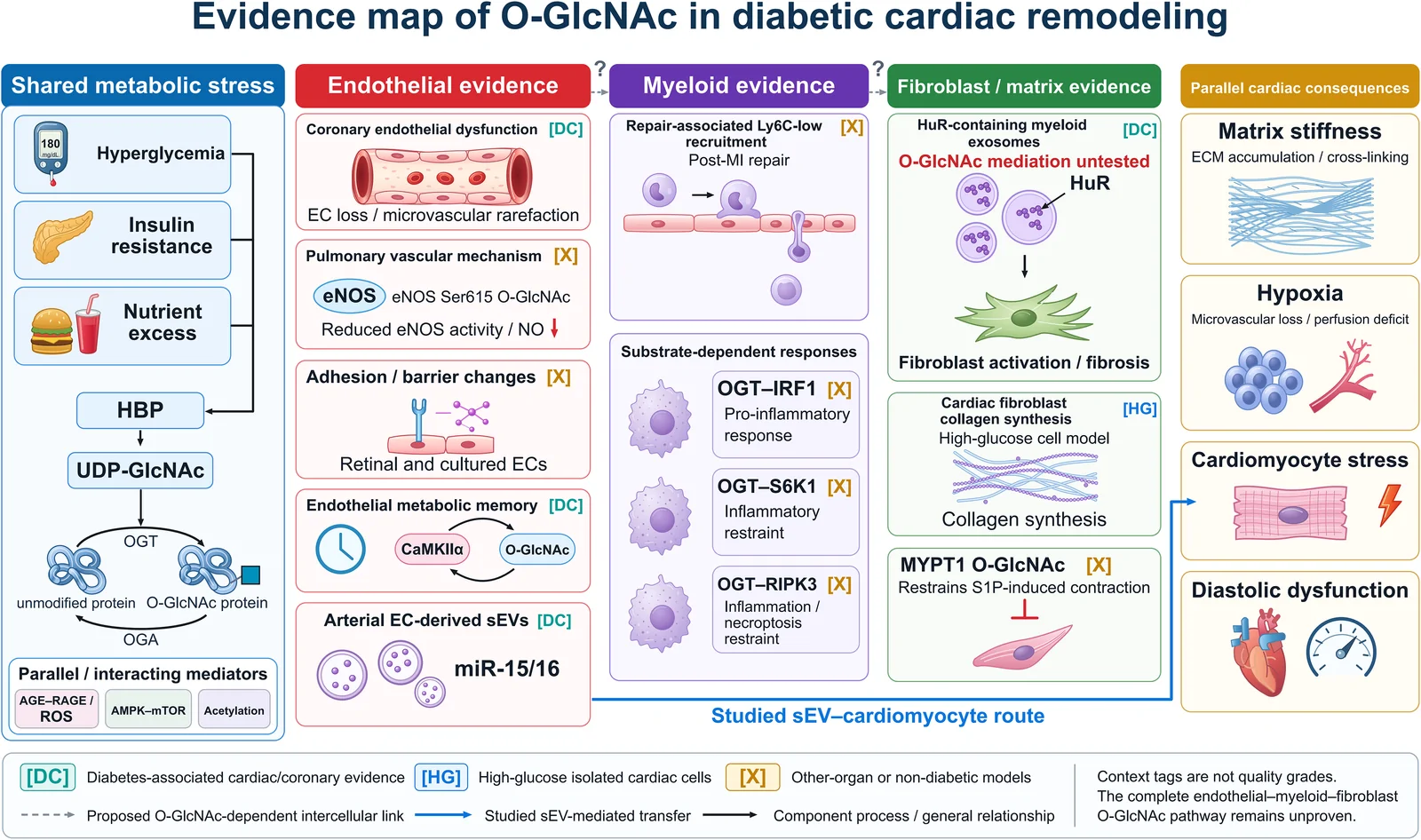
Experimental context and proposed O-GlcNAc-dependent communication in diabetic cardiac remodeling. Columns organize evidence by cell compartment, with cardiac consequences displayed in parallel. Diabetic coronary dysfunction is distinguished from the pulmonary eNOS Ser615 mechanism. Endothelial O-GlcNAc/CaMKIIα memory supports an arterial endothelial sEV miR-15/16–cardiomyocyte route after glucose normalization. This studied transfer does not establish an endothelial–macrophage–fibroblast causal chain. Repair-associated Ly6C-low recruitment in non-diabetic ischemic injury is separated from context-dependent myeloid substrate responses. Myeloid exosomal HuR–fibroblast communication has diabetic cardiac support, but its O-GlcNAc mediation remains untested. High-glucose-induced collagen synthesis in isolated cardiac fibroblasts and MYPT1-associated restraint of S1P-induced contraction represent distinct stromal findings. Context tags distinguish disease and organ relevance; they are not quality grades. Gray dashed arrows and question marks indicate proposed intercellular links whose O-GlcNAc-dependent causality remains unproven; blue arrows indicate studied sEV transfer, while black arrows indicate component processes or general relationships. AGE–RAGE, ROS, AMPK–mTOR and acetylation are shown as parallel or interacting mechanisms.

**Table 1:** Experimental design and disease relevance of selected studies.

Study and cells	Disease and organ	Tested node	Design and intervention	Main result	Main limitation
Ding et al. (6) Arterial endothelium; cardiomyocyte recipients	D/C Diabetes then normoglycemia	CaMKIIalpha–O-GlcNAc; sEV miR-15/16	Animal and cell perturbation; sEV transfer	Persistent endothelial vesicular signaling and cardiac injury	Cardiomyocyte recipients; macrophage mediation and cardiac microvascular origin not established
Masaki et al. (9) Human peripheral venous ECs	D/NC T2D participants	Global O-GlcNAc; OGT/OGA pathway	Human association; ex vivo glucose and pathway manipulation	Glucose-responsive endothelial phenotype	Venous sampling does not establish cardiac microvascular behavior
Cabrera et al. (22) Coronary ECs	D/C HFD and low-dose STZ	OGA; global O-GlcNAc	Inducible endothelial-targeted OGA overexpression; EC assays	Improved coronary microvascular function	Monocyte trafficking and macrophage mediation not tested
Si et al. (23) Coronary ECs	D/C TALLYHO T2D; coronary EC assays	OGA–O-GlcNAc; p53 abundance	Endothelial-targeted OGA overexpression; p53 inhibition; cell assays	Reduced apoptosis and improved coronary and cardiac function	No recruitment test; p53 abundance is not a site-specific modification measurement
Jing and Ma (33) RAW264.7 cells; TAC hearts	Other/C Non-diabetic pressure overload	OGT; IRF1-associated O-GlcNAc	TAC with systemic AAV9-shOGT; RAW264.7 perturbation and IRF1 rescue	Reduced cardiac dysfunction/fibrosis; altered inflammatory markers	*In vivo* knockdown not macrophage-restricted; mechanism relies on a cell line
Yang et al. (13) Myeloid cells	Other/NC HFD obesity and insulin resistance	OGT; S6K1	Myeloid OGT deletion; macrophage experiments	OGT restrained inflammation and metabolic dysfunction	No diabetic cardiac model; obesity is not automatically diabetes
Xu (38) Blood; THP-1-derived macrophages	D/NC Diabetic periodontitis; HG/LPS cells	OGT; GSDME-associated O-GlcNAc	Human expression association; cell knockdown and rescue	HG-associated GSDME/OGT changes promoted pyroptosis	Clinical data measure GSDME expression; O-GlcNAc mechanism is cellular and non-cardiac
Aguilar et al. (44) Primary cardiac fibroblasts	HG/C-cell High-glucose culture	OGA; global O-GlcNAc	Glucose challenge; adenoviral OGA expression	OGA reversed HG-associated collagen and regulatory changes	Isolated cells; no diabetic animal or immune/endothelial compartment
Pedowitz et al. (19) NIH3T3 fibroblasts	Other/NC S1P contraction	MYPT1 O-GlcNAc	Inhibitors; chemoenzymatic detection; modification-rich-region deletion	MYPT1 O-GlcNAc restrained S1P-induced contraction	Non-cardiac and non-diabetic; region deletion is not a single-site test
Li et al. (48) Cardiac fibroblasts	D/C STZ diabetic mice	LOX; O-GlcNAc not tested	Single-cell mapping; BAPN intervention; cell assays	LOX inhibition reduced fibrosis and improved function	Disease-relevant fibrosis without O-GlcNAc mediation evidence
Prakoso et al. (7) Myocardium/cardiomyocytes	D/C Diabetic human myocardium and mice	OGT, OGA; global O-GlcNAc	Human association; cardiac-targeted OGT/OGA expression	Altered structural and functional cardiac phenotypes	Does not resolve the endothelial–macrophage–fibroblast route

Design categories describe the strongest directly relevant experiment, not a numerical quality rank: human association, *in vivo* intervention, cellular mechanism or observational mapping. Human observations and cell experiments are named separately when both occur; no composite I–IV score is assigned. Disease and organ context are independent of design. D indicates diabetic participants or animals; HG indicates a high-glucose cell model without such a cohort; other indicates a different disease or metabolic context. C indicates cardiac tissue or an *in vivo* cardiac endpoint; C-cell indicates isolated cardiac cells only; NC indicates no cardiac model. A D/C label does not establish O-GlcNAc mediation. Worked example: Masaki et al. (9) combine human association and ex vivo endothelial experiments and are D/NC because the sampled cells were peripheral venous endothelium. Their human origin does not confer cardiac microvascular specificity. **Table 1** contains selected examples; Section 2.1 accounts for the full bibliography.

EC, endothelial cell; HFD, high-fat diet; STZ, streptozotocin; T2D, type 2 diabetes; TAC, transverse aortic constriction; S1P, sphingosine-1-phosphate; LOX, lysyl oxidase; BAPN, beta-aminopropionitrile; sEV, small extracellular vesicle.

## 2 O-GlcNAc as a candidate mediator of nutrient-sensitive remodeling

The hexosamine biosynthetic pathway (HBP) integrates glucose, glutamine, acetyl-CoA and uridine metabolism to generate UDP-GlcNAc. Hyperglycemia, insulin resistance and nutrient excess can alter substrate supply and OGT/OGA activity or localization. O-GlcNAc modification can then change transcription-factor occupancy, enzyme activity, protein stability, trafficking and phosphorylation (3, 5). Tissue-wide staining cannot identify the responsible cell or distinguish greater substrate modification from altered protein abundance or cellular composition.

Persistence provides a stronger rationale for the hypothesis than nutrient sensitivity alone. Endothelial O-GlcNAc signaling persisted after insulin restored normoglycemia in a diabetic model, with a CaMKIIalpha feedback circuit and small extracellular vesicles (sEVs) transmitting injury to cardiomyocytes (6). Cardiac-targeted OGT/OGA manipulations also altered diabetic cardiac structure and function, while human myocardial O-GlcNAc abundance was associated with left-ventricular dysfunction (7). These studies support functional involvement at defined steps; they do not identify O-GlcNAc as the unique coordinating mechanism.

Human immune and vascular measurements reinforce the need for compartment-specific interpretation. Leukocytes from people with diabetes had increased OGA expression and activity correlated with inflammatory markers, a circulating biomarker association that may reflect compensation rather than reduced pathway engagement (8). Fresh endothelial cells obtained by J-wire biopsy from people with type 2 diabetes also displayed an O-GlcNAc-linked glucose-responsive phenotype (9). Because these were peripheral venous cells, they cannot be assumed to reproduce cardiac microvascular behavior, but they establish patient-derived endothelial relevance.

An immunometabolic switch is used here as a functional hypothesis, not as a claim of binary or uniformly harmful behavior. Its effect must be specified by substrate, cell, timing and disease context. A small change in occupancy of a regulatory site may matter without a detectable change in total O-GlcNAc abundance. Conversely, a large bulk signal need not identify the protein controlling immune traffic. Both gain and loss of OGT activity can therefore be harmful through different substrates.

### 2.1 Competing explanations

O-GlcNAc is one candidate mediator among interacting diabetic pathways. Shared hyperglycemia, advanced glycation end products (AGEs), receptor for AGEs (RAGE) activation and reactive oxygen species (ROS) can explain parallel injury across compartments. RAGE blockade prevented cardiac dysfunction in db/db mice without testing O-GlcNAc mediation (10). These pathways also intersect: mitochondrial superoxide activated the HBP in cultured endothelial cells (11), and reciprocal OGT–AMPK regulation and OGT-dependent restraint of the mTOR effector S6K1 argue against a fixed nutrient-sensing hierarchy (12, 13). Glucose-derived acetyl-CoA provides another route to transcriptional regulation through histone acetylation (14). The endothelial persistence experiment (6) therefore supplies a specific rationale for O-GlcNAc beyond nutrient responsiveness, but it does not exclude AGE–RAGE, redox or chromatin mechanisms that could also outlast hyperglycemia. A coordinating role requires evidence that O-GlcNAc perturbation changes an identified donor-cell signal and recipient response at matched glycemia. Present findings support candidate mediation and persistence, rather than dominance over these alternative or parallel mechanisms.

We audited all 58 cited references: 48 were peer-reviewed biological or clinical original reports, three were primary methods papers and seven were reviews. Among the 48 biological/clinical reports, 23 included confirmed diabetic participants or animals, three used only high-glucose cell models, 17 examined other settings and five had unresolved diabetes inclusion in human tissue or reused datasets. Independently, 29 studied cardiac tissue, coronary vessels or cardiac outcomes, one used isolated cardiac fibroblasts only and 18 lacked direct cardiac context. Seventeen combined confirmed diabetes inclusion and direct cardiac context; one was a mixed HFpEF cohort with diabetic comorbidity, leaving 16 with a diabetes-directed cardiac design. These counts characterize this bibliography, not O-GlcNAc mediation or validation of the proposed sequence. **Table 1** presents 11 selected studies.

### 2.2 Measurement and molecular attribution

Measurement limits the resolution of these claims. RL2 and CTD110.6 pan-O-GlcNAc antibodies detect overlapping but unequal protein populations, and cardiac extraction conditions alter the apparent signal (15). CTD110.6 can also recognize truncated N-linked glycans under severe nutrient deprivation; RL2 did not show this cross-reactivity in the same validation study (16). Concordant antibody results, OGT/OGA perturbation and appropriate glycan-removal controls are therefore more informative than one bulk blot. Neither immunoreactivity nor immunoprecipitation alone identifies a modified residue or its occupancy. Chemoenzymatic tagging and click-based enrichment improve substrate recovery, but per-O-acetylated metabolic sugar reporters can generate artificial cysteine S-glycosylation (17). Site-localizing mass spectrometry with functional mutagenesis and rescue strengthens residue attribution. eNOS Ser615 was examined using LC–MS/MS of recombinant protein and mutagenesis in expression systems (18). MYPT1 was supported by chemoenzymatic enrichment, PEG mass-shift measurements and deletion-mutant replacement, but removing a serine/threonine-rich region does not isolate one causal glycosite (19). Sp1–ICAM-1 is therefore discussed as a substrate/pathway mechanism rather than an equivalently residue-resolved example (20). None of these measurements alone validates the same modified site in diabetic cardiac endothelium or fibroblasts.

## 3 Endothelial O-GlcNAc reprogramming and metabolic memory

Cardiac microvascular endothelial cells control perfusion, leukocyte capture, barrier function and paracrine support. Single-cell and spatial profiling of the coronary microcirculation and myocardium in db/db mice identifies endothelial heterogeneity and vascular remodeling programs relevant to early coronary microvascular disease (21). These maps do not prove O-GlcNAc causality, but they define the murine tissue context in which an endothelial nutrient-sensing program must operate.

Diabetes-specific intervention studies provide causal vascular evidence. Endothelial-targeted OGA overexpression restored coronary microvascular function in high-fat-diet plus low-dose streptozotocin mice (22). In another type 2 diabetic model, excess O-GlcNAcylation was linked to p53 accumulation, coronary endothelial apoptosis, microvascular rarefaction and cardiac dysfunction; inducible endothelial OGA overexpression and p53 perturbation supported the mechanism (23). Endothelial-targeted OGA overexpression also reversed coronary endothelial dysfunction in type 1 diabetic mice (24). These experiments support an endothelial contribution but did not establish macrophage recruitment as the mediator of the cardiac phenotype.

Candidate immune-recruitment mechanisms have less direct cardiac support. High glucose altered a miR-200a/b–OGT circuit and inflammatory signaling in aortic endothelial cells (25). Retinal studies linked Sp1 O-GlcNAc modification to ICAM-1 induction (20) and O-GlcNAc inhibition to improved connexin-43 expression and barrier properties (26). An eNOS Ser615 mechanism was resolved in pulmonary vascular research (18). These findings nominate adhesion, nitric-oxide and junctional pathways for cardiac testing; they do not establish those mechanisms in coronary microvascular endothelium. Protein-level Sp1 attribution also has a different evidentiary resolution from an individually mapped eNOS site.

Glycemic volatility may further intensify endothelial reprogramming. Recurrent hypoglycemia in diabetic rats increased eNOS O-GlcNAcylation, reduced activating phosphorylation and worsened endothelium-dependent vasodilation in non-coronary vessels (27). The finding cautions against equating lower mean glucose with normalized endothelial signaling. Hyperglycemia-dependent O-GlcNAc modification of CaMKII has also been detected across human, rat and mouse cardiac preparations, linking diabetic nutrient stress to electrical and signaling instability (28); its direct endothelial contribution remains uncertain.

The CaMKIIalpha–sEV study identified arterial endothelial cells as a source of miR-15/16-enriched vesicles and cardiomyocytes as recipients after glycemic normalization (6). It therefore supports persistent endothelial-to-myocyte communication; it should not be relabeled as a demonstrated cardiac microvascular endothelial-to-macrophage mechanism. In a non-diabetic mouse model of HFpEF, endothelial mitochondrial dissociation of hexokinase-1 increased protein O-GlcNAcylation and contributed to disease (29). This cardiac precedent supports mechanistic plausibility across metabolic states without establishing diabetes-specific immune mediation.

Vascular-bed identity matters. Coronary capillary endothelial cells experience mechanical load and close metabolic exchange with cardiomyocytes and pericytes; peripheral venous or retinal cells may respond differently to the same glucose history. Loss of vasodilation or barrier integrity alone also does not demonstrate an immune-instructive state. Such an interpretation requires measurements of adhesion under flow, transendothelial migration or defined paracrine responses in recipient cells.

Integrated chromatin and transcriptome analyses identify coordinated endothelial and fibroblast states in diabetic cardiomyopathy (30). Diabetic mouse atlases further identify altered ligand–receptor networks involving endothelium, macrophages and fibroblasts (31) and broader non-myocyte remodeling (32). These observational maps localize candidate interactions, but inferred ligand–receptor expression does not establish O-GlcNAc-dependent communication.

## 4 Endothelial–macrophage crosstalk and state transitions

In the proposed network, endothelial adhesion molecules, chemokines, nitric-oxide availability and vesicle cargo influence monocyte arrest, extravasation and persistence. Resident macrophages may respond without recruitment, so an inflammatory myocardial signature does not establish a blood-monocyte source. The analysis below separates myeloid O-GlcNAc mechanisms from diabetic cardiac macrophage functions to identify which connections remain to be tested.

Macrophage-intrinsic O-GlcNAc effects are conditional. OGT-dependent modification regulated an IRF1-associated inflammatory program in a non-diabetic pressure-overload heart-failure study with RAW264.7 mechanistic experiments (33). Under overnutrition, myeloid OGT instead restrained S6K1 signaling, inflammation and systemic metabolic dysfunction (13). OGT-dependent modification of RIPK3 at T467 limited necroptosis and inflammatory activation in innate immune models (34). These divergent results argue against predicting macrophage behavior from total OGT activity alone.

Other substrates reinforce this context dependence. O-GlcNAc-modified NLRX1 interacted with IKKalpha to regulate IL-1beta expression (35); chronic O-GlcNAc elevation reduced nitric-oxide production but deregulated selected cytokines rather than imposing one activation state (36). FTO modification promoted TRIM21-mediated FTO degradation and inflammatory negative feedback (37). A periodontitis study combined diabetic patient blood observations with high-glucose/LPS experiments in THP-1-derived macrophages, implicating GSDME O-GlcNAcylation in pyroptosis (38). That mechanism was resolved in cultured macrophages, not in the diabetic heart.

Recruitment itself may also depend on O-GlcNAc context. Glucosamine enhanced post-ischemic cardiac recovery by recruiting Ly6C-low monocytes through STAT1- and O-GlcNAc-dependent signaling (39). Local thiamet-G delivery after myocardial infarction likewise shifted myeloid repair through STAT6 O-GlcNAcylation (40). Both studies are non-diabetic injury models and used pharmacologic elevation, so they cannot establish that chronic diabetic endothelial O-GlcNAc recruits the same cells or yields the same outcome. They do show that O-GlcNAc can alter cardiac monocyte entry and reparative-state transitions *in vivo*.

Macrophage states should be described by origin, metabolic program, signaling modules and function, rather than a binary M1/M2 classification. In diabetic cardiac models, macrophage-derived S100A9 disrupted cardiomyocyte mitochondrial quality control through STAT3 (41), and Dectin-1 deficiency reduced macrophage-mediated inflammation and cardiac injury (42). These studies establish disease-relevant myeloid effects without testing O-GlcNAc mediation.

Bidirectional feedback is biologically plausible: macrophage cytokines and reactive species could aggravate endothelial nitric-oxide loss, barrier disruption and chemokine production, while stiffness and hypoxia could sustain both vascular and myeloid activation. The endothelial memory study provides a candidate source of persistent vesicular signals (6). Whether cardiac macrophages respond to this cargo, and how it contributes to feedback, require experimental resolution.

A diabetes-specific downstream communication pathway is supported without direct testing of O-GlcNAc mediation: increased monocyte/macrophage exosomal HuR was linked to cardiac inflammation, fibroblast activation and fibrosis, and targeting this axis attenuated those outcomes (43). Thus, the proposed network combines evidence for endothelial persistence, conditional myeloid substrate effects and diabetic myeloid-to-fibroblast communication. HuR-dependent fibrosis cannot itself establish that O-GlcNAc links these steps.

Persistent activation also requires precise terminology. Endothelial metabolic memory is supported after glucose normalization (6), whereas trained immunity would require durable myeloid reprogramming after stimulus withdrawal and rest. Continued activation in a diabetic milieu, prolonged cell retention and progenitor-level memory are distinct explanations. They should not be conflated without lineage and persistence measurements.

## 5 Fibrotic reprogramming as a downstream multicellular state

Fibrosis reflects matrix production, contraction, turnover and communication between stromal and immune cells. High glucose increased protein O-GlcNAcylation and collagen synthesis in primary cardiac fibroblasts (44), providing cardiac cell evidence under a diabetic stimulus rather than an intact diabetic-heart mechanism. MYPT1 O-GlcNAc modification restrained sphingosine-1-phosphate-driven contraction in non-cardiac fibroblasts (19), and OGT influenced collagen deposition and resolution in pulmonary fibrosis (45). These latter studies identify candidate mechanisms whose cardiac transfer remains untested.

Human cardiac single-cell and multiomic studies show that immune-fibroblast communication is active in acute infarction and chronic heart failure (46). Cross-tissue work identifies SPP1-positive macrophages that drive myofibroblast activation through a CXCL4-dependent circuit (47). Neither study is diabetes-specific or O-GlcNAc-resolved. In diabetic mice, however, single-cell analysis identified a Lox-positive fibroblast subset, and LOX inhibition reduced fibrosis and improved cardiac function (48). Together with exosomal HuR signaling from diabetic macrophages, these data frame fibrosis as a multicellular output while preserving the uncertainty over which fibroblast O-GlcNAc substrates integrate immune cues.

Co-culture and conditioned-medium findings must distinguish direct contact, soluble mediators and vesicles. *In vivo*, fibroblast expansion may follow endothelial injury, respond directly to the diabetic milieu or feedback through matrix-bound growth factors. Assigning this response to an O-GlcNAc substrate requires perturbation of that substrate and measurement of matrix synthesis, contraction and degradation, rather than inference from increased collagen alone.

Matrix accumulation feeds back on the initiating compartments. Increased stiffness changes integrin and cytoskeletal signaling in fibroblasts, endothelial cells and macrophages; capillary rarefaction increases hypoxia; and reduced compliance elevates diastolic load. Matrix composition is also instructive: cross-linking, latent growth-factor release and impaired collagen turnover can maintain inflammatory neighborhoods after the initiating glucose signal has subsided. Fibrosis should therefore be measured by organization, mechanics and reversibility, not hydroxyproline or collagen area alone.

Cardiomyocyte stress can amplify the network. In cardiomyocyte-specific OGT-transgenic mice, increased O-GlcNAcylation was reported with dilated cardiomyopathy, ventricular arrhythmias and premature death (49). Chronic GFAT1-driven HBP activation triggered remodeling (50), and prolonged augmented O-GlcNAcylation worsened hypoxia-associated fibrosis (51). Conversely, HDAC4 O-GlcNAcylation protected the diabetic heart (52), and Tisp40–HBP signaling protected against acute ischemia–reperfusion injury in male mice (53). These models support duration- and substrate-dependent cardiac effects without demonstrating the proposed immune route.

An intervention that globally lowers O-GlcNAc might therefore remove adaptive myocardial or immune substrates while reducing chronic endothelial or fibroblast signaling. The relevant fibrotic endpoint is whether a defined perturbation interrupts endothelial activation, myeloid communication, fibroblast state conversion and organ stiffness without impairing acute stress tolerance.

## 6 Therapeutic implications and future directions

The pathway offers targets at several resolutions. HBP inhibition at GFAT could reduce UDP-GlcNAc supply, OGT inhibition could block addition, and OGA activation could accelerate removal. Each global strategy is intrinsically blunt because OGT and OGA are essential, widely expressed and shared by protective and pathogenic substrates. Metabolite-dependent post-translational modifications also interact with phosphorylation, acetylation and redox signaling, making pathway-wide inhibition difficult to interpret (54). Immune-cell reviews reach the same conclusion: O-GlcNAc output depends on activation context and substrate rather than total abundance alone (55).

Sex is an unresolved boundary of the intervention evidence. The coronary OGA studies used male mice (22, 24), as did the pressure-overload Gfat1 and Tisp40 ischemia–reperfusion experiments (50, 53). These cohorts cannot establish whether responses generalize to females. In non-diabetic perfused mouse hearts, females showed higher O-GlcNAcylation and OGT activity than age-matched males under the conditions examined (56). This supports sex-dependent pathway regulation without establishing its direction in diabetic endothelial–immune remodeling. Future studies should include both sexes, test sex-by-intervention interactions, and report age and relevant hormonal status rather than assume that a male-derived therapeutic window applies across sexes.

In a diabetic model, inducibly perturb endothelial OGT/OGA after disease onset and match glucose and insulin exposure, blood pressure and systemic metabolic status between groups, with endothelial viability and perfusion controls. Establish temporal order by measuring endothelial cargo and adhesion, fate-mapped recruited monocytes versus resident macrophages, and subsequent fibroblast states and spatial matrix mechanics. Identify the transmitted ligand or sEV cargo, test recipient-pathway necessity, and restore that cargo to test rescue. Persistence after normoglycemia and ex vivo rest should be distinguished from continued stimulation. Measure AGE–RAGE, redox and AMPK/mTOR activity and acetylation, then use targeted comparator perturbations to distinguish mediation from parallel responses. Finally, test a validated substrate-site rescue with expression and phosphorylation controls. A cargo-dependent myeloid and fibrotic response would support the integrating hypothesis; preserved myeloid recruitment and fibrosis despite verified endothelial target engagement would weaken it. Fibroblast endpoints should include contraction and collagen turnover as well as synthesis.

Timing and clinical attribution remain important. A mouse study found that transiently increasing cardiomyocyte O-GlcNAc enhanced susceptibility to subsequent pressure-overload remodeling (57). SGLT2 inhibitors and GLP-1 receptor agonists should be described as acting through O-GlcNAc-mediated crosstalk only when direct mediator experiments establish that route. Human HFpEF myocardium shows remodeling of glycolytic and ancillary pathways in a population enriched for obesity and diabetes (58), but linking those measurements to cell-specific substrates requires further work. The immediate therapeutic objective is to identify which persistent communication steps depend on O-GlcNAc while preserving adaptive myocardial and immune responses.
